# High Plasma tPAPAI-1C Levels May Be Related to a Poor Prognosis in Patients with Severe or Critical COVID-19: A Single-Center Retrospective Study

**DOI:** 10.3390/jcm12052019

**Published:** 2023-03-03

**Authors:** Kunihiro Shirai, Michiko Ishikawa, Tomoyuki Kobayashi, Kiyoko Sato, Hiromoto Murakami, Keisuke Kohama, Naomi Manbo, Kana Hasegawa, Junichi Hirata

**Affiliations:** 1 Department of Emergency, Disaster and Critical Care Medicine, Hyogo Medical University, Nishinomiya 663-8501, Japan; 2 Department of Pediatrics, Hyogo Medical University, Nishinomiya 663-8501, Japan

**Keywords:** COVID-19, tPAPAI-1C, coagulopathy, fibrinolysis, endothelial damage

## Abstract

Severe novel coronavirus disease 2019 (COVID-19) patients have a high incidence of thrombotic complications and mortality. The pathophysiology of coagulopathy involves fibrinolytic system impairment and vascular endothelial damage. This study examined coagulation and fibrinolytic markers as outcome predictors. In an observational study of 164 COVID-19 patients admitted to our emergency intensive care unit, hematological parameters on days 1, 3, 5, and 7 were retrospectively compared between survivors and nonsurvivors. Nonsurvivors had a higher APACHE II score, SOFA score, and age than survivors. Nonsurvivors also had a significantly lower platelet count and significantly higher plasmin/α2plasmin inhibitor complex (PIC), tissue plasminogen activator/plasminogen activator inhibitor-1 complex (tPAPAI-1C), D-dimer, and fibrin/fibrinogen degradation product (FDP) levels than survivors throughout the measurement period. The 7-day maximum or minimum values of the tPAPAI-1C, FDP, and D-dimer levels were significantly higher in nonsurvivors. A multivariate logistic regression analysis showed that the maximum tPAPAI-1C (OR = 1.034; 95% CI,1.014–1.061; *p* = 0.0041) was an independent factor affecting mortality, with an area under the curve (AUC) of 0.713 (optimum cut-off of 51 ng/mL; sensitivity, 69.2%; and specificity, 68.4%). COVID-19 patients with poor outcomes exhibit exacerbated coagulopathy with fibrinolysis inhibition and endothelial damage. Consequently, plasma tPAPAI-1C might be a useful predictor of the prognosis in patients with severe or critical COVID-19.

## 1. Introduction

The novel coronavirus disease 2019 (COVID-19) caused by severe acute respiratory syndrome coronavirus 2 (SARS-CoV-2) has spread throughout the world. Most cases are mild or asymptomatic, but around 5% of patients are admitted to an intensive care unit (ICU) because of a severe or critical condition [1]. In recent systematic reviews, 76.1–85.0% of patients admitted to the ICU because of COVID-19 were diagnosed as having acute respiratory distress syndrome (ARDS), and 58.0–67.7% of admitted patients received invasive mechanical ventilation, 28.0–65.9% received vasopressor therapy, and 16.6–16.9% received renal replacement therapy (95% CI, 12.1–22.2); the ICU mortality rate was 28.1–32.3% [2,3].

Patients admitted to the ICU because of severe or critical COVID-19 were characterized by the dysregulation of their immune response, a so-called “cytokine storm,” resulting in extremely high levels of inflammatory cytokines and leading to vascular endothelial dysfunction and the dysregulation of coagulation systems [4,5,6,7,8]. In particular, coagulopathy is deeply associated with a worsening prognosis. In fact, critically ill patients with coagulopathy had high incidences of thrombotic complications such as proximal deep vein thrombosis (DVT, 14%; ultrasound screening for DVT, 38%) and clinically relevant pulmonary embolism (8%) [9,10,11,12]. A recent study indicated that 76.1% of nonsurvivors and 0.6% of survivors developed overt disseminated intravascular coagulation (DIC) [13]. An elevated plasma D-dimer level is reportedly associated with disease severity and mortality in COVID-19 patients [13,14,15,16]. However, the range of “high” was within the normal limits. To predict disease severity, an easily understandable marker of the dysfunction of vascular endothelia or coagulation systems is needed.

While COVID-19-associated coagulopathy plays a pivotal role in the progression of clinical manifestations, the impairments of the fibrinolytic system and vascular endothelium that affect coagulopathy and the changes in biomarkers over time have remained unclear. The purpose of the present study was to identify coagulation and fibrinolytic parameters that can be easily obtained using blood chemistry tests and that are significantly associated with hospital outcome among COVID-19 patients.

## 2. Materials and Methods

### 2.1. Patients

This retrospective observation study was conducted at the Emergency and Critical Care Center of the Hyogo Medical University Hospital in Japan from March 2020 to March 2022. The Emergency and Critical Care Center of the Hyogo Medical University Hospital mainly treats severe and critical cases, according to the criteria of the World Health Organization’s clinical management guidance [17]. This study was approved by the institutional ethics board of the Hyogo Medical University Hospital (#4202). We published details of our study on our website and obtained informed consent from the patients based on the opt-out model (URL: https://www.hyo-med.ac.jp/research/committee/ethics/optout/ (accessed on 29 September 2022)). All consecutive patients who were more than 18 years old at the time of their admittance to our center’s ICU and who had a diagnosis of severe or critical COVID-19 were enrolled in this study. COVID-19 was confirmed by laboratory testing using the polymerase chain reaction (PCR) method, and pneumonia was confirmed based on a computed tomography examination. Hematological test results were obtained during routine clinical laboratory testing. The study exclusion criteria were hematological disorders, cirrhosis, current malignancy, and the withholding or withdrawal of life-sustaining treatments. We evaluated data for nonsurvivors, compared with that of survivors, among patients with severe or critical COVID-19.

### 2.2. Data Collection

All demographic, treatment, laboratory, and outcome data were extracted from the electronic patient medical records of the emergency ICU, Hyogo Medical University Hospital. The following items were recorded for each patient: sex, body mass index (BMI), history of smoking, comorbidity, use of a high-flow nasal canal, mechanical ventilation, extracorporeal membrane oxygenation, renal replacement therapy, prone position, corticosteroids treatment, tocilizumab treatment, heparin treatment, and antibiotics treatment. The following hematological parameters assessed at admission (day 1) and on days 3, 5, and 7 were also examined: white blood cell (WBC) count, c-reactive protein (CRP), platelet (PLT) count, prothrombin time-international normalized ratio (PT-INR), activated partial thromboplastin time (APTT), fibrinogen, soluble fibrin (SF), thrombin/antithrombin III complex (TAT), von Willebrand Factor (vWF), α2plasmin inhibitor (α2PI), plasmin/α2plasmin inhibitor complex (PIC), D-dimer, fibrin/fibrinogen degradation products (FDP), antithrombin activity (AT activity), thrombomodulin (TM), protein C (PC), protein S (PS), and tissue plasminogen activator/plasminogen activator inhibitor-1 complex (tPAPAI-1C). Because some markers (SF, TAT, vWF, α2PI, PIC, FDP, AT activity, TM, PC, PS, and tPAPAI-1C) are not routinely measured in laboratories, data on these parameters could not be obtained on holidays. Disseminated intravascular coagulation (DIC) was defined using the diagnostic criteria of the International Society on Thrombosis and Haemostasis (ISTH) for an overt DIC score of 5 points or more. The severity of the patient’s underlying medical condition was assessed using their Acute Physiology and Chronic Health Evaluation II (APACHE II) score and their Sequential Organ Failure Assessment (SOFA) score [18,19]. The Charlson comorbidity index was used to evaluate the risk of death arising from comorbidity [20,21].

### 2.3. Statistical Analysis

Data were analyzed using JMP version 15.0 (SAS Institute, Cary, NC, USA). Continuous variables were presented as the mean ± standard deviation for normal distributions and as the median and interquartile range (IQR) for nonnormal distributions. Categorical variables were expressed as numbers and the percentages of individuals in each category. To compare survivors and nonsurvivors, the Student t-test or Wilcoxon signed-rank test was used as a parametric analysis, and the chi-square test or Fisher exact test was used as a nonparametric analysis. Differences among measurement days were evaluated using an analysis of variance. To determine the independent risk factors for mortality, univariate and stepwise multiple logistic regressions were performed. The sensitivity and specificity of the strongest expected risk factor were analyzed using receiver operating characteristic (ROC) curves, and the area under the curve (AUC) was calculated. All tests were two-tailed, and *p* values < 0.05 were considered statistically significant.

## 3. Results

### 3.1. Demographic and Clinical Characteristics

One hundred and seventy patients were diagnosed as having severe or critical COVID-19 and were admitted to our ICU. A total of 6 patients were excluded based on the study’s exclusion criteria, and 164 patients were enrolled. The mortality rate was 20.1% (131 survivors and 33 nonsurvivors). The baseline and clinical characteristics of the survivors and nonsurvivors are shown in Table 1. The median APACHE II score and the SOFA score were 11 (IQR:7–17) and 3 (IQR:2–6), respectively. The nonsurvivors were significantly older and had a higher Charlson comorbidity index, severity, SOFA score, APACHE II score, and DIC score than the survivors. No significant differences in sex or BMI were seen between the survivors and nonsurvivors. Around half of the patients had a history of smoking, but this parameter had no effect on mortality.

Table 2 shows the comorbid conditions in the patients. Hypertension (47.6%) and diabetes (40.2%) were the most common comorbidities. However, neither of these comorbid conditions nor any of the others had an effect on mortality. 

Table 3 shows the patient treatments. The use of a high-flow nasal canal, corticosteroids, tocilizumab, and heparin did not differ significantly between the survivors and the nonsurvivors. The use of remdesivir was more frequent among survivors (55.7%) than among nonsurvivors (33.3%). Mechanical ventilation, extracorporeal membrane oxygenation, renal replacement therapy, and a prone position were used more frequently among nonsurvivors than among survivors. In addition, significantly more nonsurvivors than survivors received antibiotics for secondary infections. This section may be divided by subheadings. It should provide a concise and precise description of the experimental results, their interpretation, as well as the experimental conclusions that can be drawn.

### 3.2. Hematological Parameters

**Figure 1:**
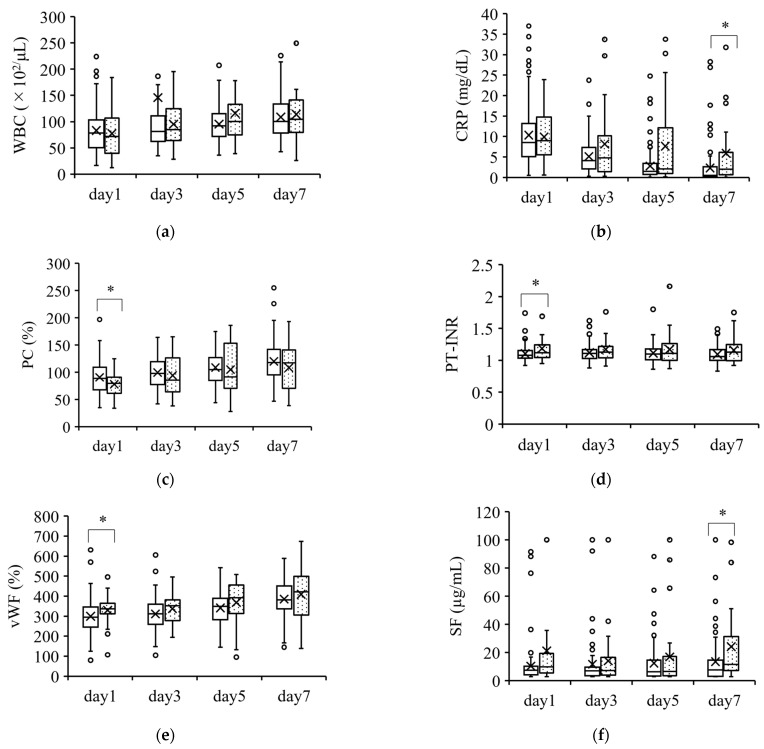
Coagulation parameters with minimal differences between survivors and nonsurvivors. Parameters with a minimal ability to predict severity are shown in (**a**–**f**). The boxplots show the median and interquartile ranges; “X” indicates the mean value. The circles show individual outliers. The white boxes denote survivors and the boxes with the dot pattern denote nonsurvivors. Data in nonsurvivors/survivors: (**a**) white blood cell count (WBC): 33/132 on day 1, 33/131 on day 3, 33/130 on day 5, and 31/115 on day 7; (**b**) serum C-reactive protein (CRP): 33/129 on day 1, 33/128 on day 3, 32/128 in day 5, and 31/112 on day 7; (**c**) serum protein C (PC): 26/103 on day 1, 23/89 on day 3, 25/86 on day 5, and 27/91 on day 7; (**d**) serum prothrombin time–international normalized ratio (PT-INR): 33/129 on day 1, 33/129 on day 3, 33/128 on day 5, and 31/112 on day 7; (**e**) serum von Willebrand factor (vWF): 26/103 on day 1, 23/88 on day 3, 24/86 on day 5, and 26/90 on day 7; and (**f**) serum soluble fibrin (SF): 25/102 on day 1, 23/88 on day 3, 25/86 on day 5, and 27/91 on day 7. The Student t-test or Wilcoxon signed-rank test was used for comparisons between survivors and nonsurvivors. * *p* < 0.05.

**Figure 2:**
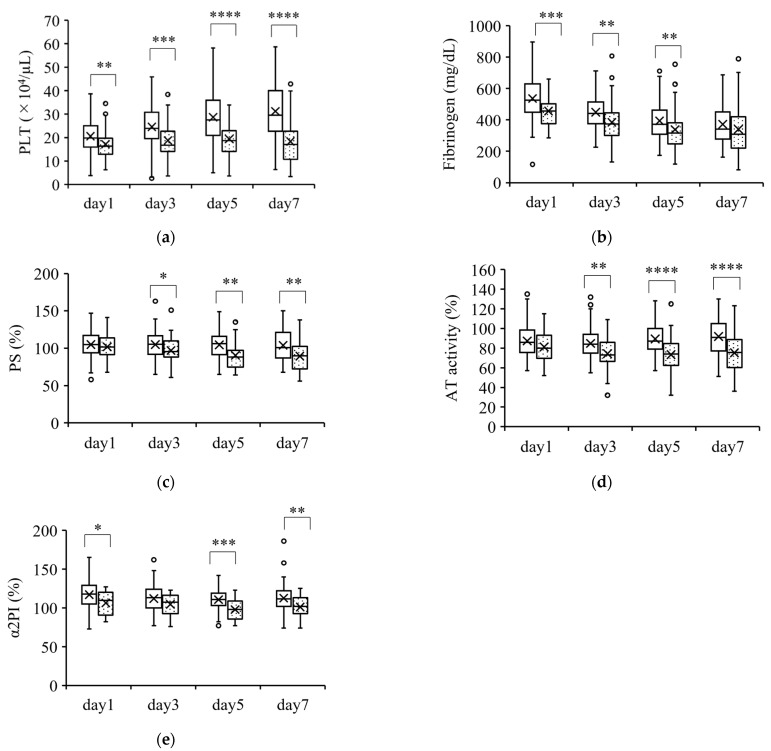
Coagulation parameters that were lower in nonsurvivors than in survivors. Parameters that were significantly lower in nonsurvivors than in survivors over the course of 7 days are shown in (**a**–**e**). The boxplots show the median and interquartile range; “X” indicates the mean value. The circles show individual outliers. The white boxes denote survivors and the boxes with the dot pattern denote nonsurvivors. Data in nonsurvivors/survivors: (**a**) platelet count (PLT): 33/129 on day 1, 33/129 on day 3, 33/127 on day 5, and 31/111 on day 7; (**b**) plasma fibrinogen: 33/129 on day 1, 33/129 on day 3, 33/127 on day 5, and 31/109 on day 7; (**c**) serum protein S (PS): 26/103 on day 1, 23/89 on day 3, 25/86 on day 5, and 27/91 on day 7; (**d**) plasma antithrombin activity (AT activity): 33/129 on day 1, 33/126 on day 3, 33/127 on day 5, and 31/110 on day 7; and (**e**) plasma α2 plasmin inhibitor (α2PI) 22/96 on day 1, 21/88 on day 3, 23/86 on day 5, and 24/91 on day 7. The Student t-test or Wilcoxon signed-rank test was used for comparisons between survivors and nonsurvivors. * *p* < 0.05, ** *p* < 0.01, *** *p* < 0.001, **** *p* < 0.0001.

**Figure 3:**
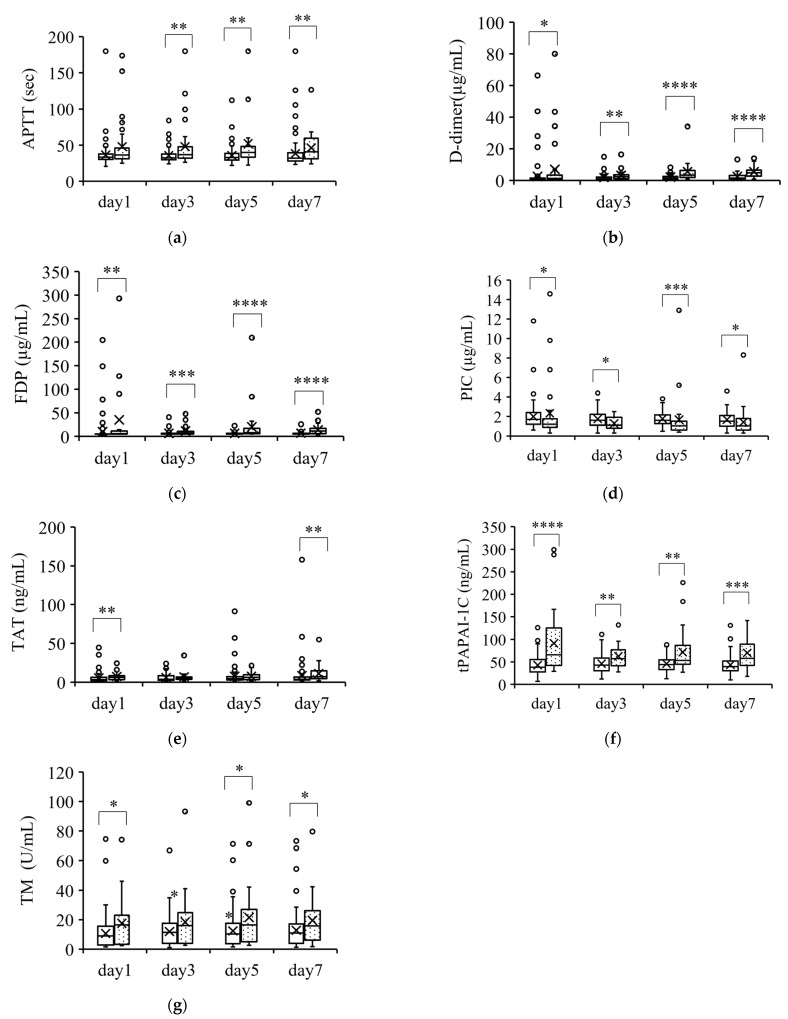
Coagulation parameters that were higher in nonsurvivors than in survivors. Parameters that were significantly higher in nonsurvivors than in survivors over the course of 7 days are shown in (**a**–**e**). The boxplots show the median and interquartile range; “X” indicates the mean value. The circles show individual outliers. The white boxes denote survivors and the boxes with the dot pattern denote nonsurvivors. Data in nonsurvivors/survivors: (**a**) plasma-activated partial thromboplastin time (APTT): 33/129 on day 1, 33/129 on day 3, 33/128 on day 5, and 31/111 on day 7; (**b**) serum D-dimer: 33/129 on day 1, 33/129 on day 3, 33/127 on day 5, and 31/111 on day 7; (**c**) plasma fibrin degradation products (FDP): 28/124 on day 1, 30/124 on day 3, 30/121 on day 5, and 31/111 on day 7; (**d**) plasmin/α2plasmin inhibitor complex (PIC): 26/103 on day 1, 23/89 on day 3, 25/86 on day 5, and 27/91 on day 7; (**e**) plasma thrombin/antithrombin III complex (TAT): 26/123 on day 1, 23/89 on day 3, 25/86 on day 5, and 28/91 on day 7; (**f**) plasma total plasmin activator and plasmin activator inhibitor-1 complex (tPAPAI-1C): 26/103 on day 1, 23/89 on day 3, 25/86 on day 5, and 27/92 on day 7; and (**g**) plasma thrombomodulin (TM): 25/101 on day 1, 23/89 on day 3, 23/84 on day 5, and 26/90 on day 7. The Student t-test or Wilcoxon signed-rank test was used for comparisons between survivors and nonsurvivors. * *p* < 0.05, ** *p* < 0.01, *** *p* < 0.001, **** *p* < 0.0001. First item.

Hematological parameters on days 1, 3, 5, and 7 were compared between survivors and nonsurvivors in Figure 1, Figure 2 and Figure 3. Figure 1 shows the parameters with minimal differences between survivors and nonsurvivors over the course of 7 days. The CRP and SF were significantly different on day 7, while the PC, PT-INR, and vWF were significantly different on day 1. However, these differences were of minimal value for predicting patient severity.

Figure 2 shows the parameters that were significantly lower in nonsurvivors than in survivors. These parameters (PLT count, fibrinogen, PS, AT activity, and α2PI) were significantly lower in nonsurvivors than in survivors at almost all the measurement points. However, the changes in these parameters were within the normal ranges. 

Figure 3 shows the parameters that were significantly higher in nonsurvivors than in survivors. These parameters (APTT, D-dimer, FDP, PIC, TAT, tPAPAI-1C, and TM) were significantly higher in nonsurvivors than in survivors at almost all the measurement points. However, the changes in these parameters were within the normal ranges except for the change in tPAPAI-1C.

### 3.3. tPAPAI-1 May Be a Prognostic Marker of COVID-19 Severity

To evaluate prognostic markers, we performed a multivariate logistic regression. Age, sex, and BMI are patient characteristics that might affect the pathology of COVID-19. The APACHE II and SOFA scores reflect the clinical severity of the patients’ conditions. In our study, the plasma tPAPAI-1C levels were above the normal range throughout the 7 days of observation, especially among nonsurvivors. Therefore, we focused on tPAPAI-1 (to evaluate fibrinolysis and endothelial dysfunction), AT activity (to evaluate extravasation, such as edema), and the D-dimer level (to evaluate coagulation disorders) as predictors of COVID-19 severity. We used the maximum or minimum data points during the 7-day observation period after admission for these hemorrhagic parameters (Table 4). 

**Figure 4:**
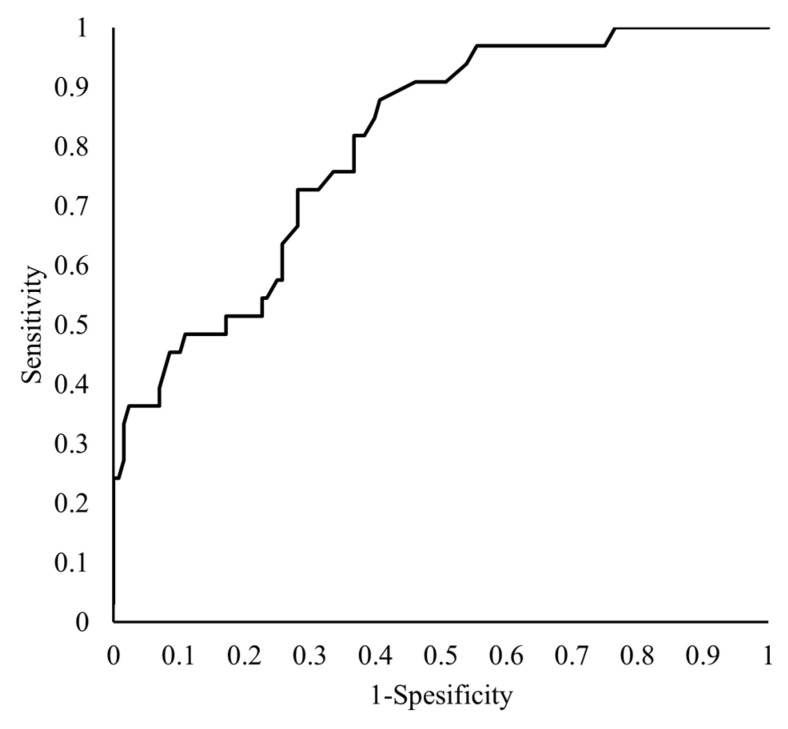
Receiver operating characteristic curves of tPAPAI-1C for predicting mortality. The vertical axis shows the “Sensitivity,” while the horizontal axis shows the “1-Specificity” of the tPAPAI-1C as a predictor of patient mortality.

The tPAPAI-1C (OR = 1.034; 95% CI,1.014–1.061; *p* = 0.0041) was an independent factor affecting the mortality of patients with COVID-19 (Table 3). The ROC curve for tPAPAI-1C is shown in Figure 4. The AUC for tPAPAI-1C was 0.713, and the optimum cut-off value was 51 ng/mL, with a sensitivity of 69.2% and a specificity of 68.4% (Figure 4).

## 4. Discussion

In the present study, nonsurvivors of COVID-19 were older and had higher severity scores, such as SOFA or APACHE II; they also required several treatments to rescue their severe conditions more often than the survivors. The PLT, PIC, tPAPAI-1C, D-dimer and FDP parameters were also worse in the nonsurvivors on days 1, 3, 5 and 7, compared with those in the survivors. In addition, when the maximum or minimum values of each hematological parameter were compared, nonsurvivors had a reduced PLT count and prolonged clotting time, enhanced coagulation, enhanced fibrinolysis, and increased vascular endothelial dysfunction. However, only plasma tPAPAI-1C levels were clearly higher than values in the normal range. In the multivariate analysis, the maximum tPAPAI-1C value during a 7-day observation period after hospital admission was identified as a strong predictor of mortality. 

A positive correlation has been reported to exist between age and the APACHE II score (R^2^ = 0.114, *p* < 0.01, data not shown), presumably because age is one of the items included in the calculation of the APACHE II score. However, we believe that age has little influence on organ dysfunction, as no correlation has been shown between the SOFA score and age (R^2^ = 0.024, data not shown); we speculated other causes for the higher SOFA scores in the nonsurvivors as compared with the survivors. We also checked the correlations between the blood parameters and age (data not shown). None of the parameters, including the plasma tPAPAI-1C, showed any correlation with the age, except plasma AT activity (day 3, R^2^ = 0.134, *p* < 0.01; day 5, R^2^ = 0.208, *p* < 0.01; and day 7, R^2^ = 0.137, *p* < 0.01), plasma α2PI (day 3, R^2^ = 0.134, *p* < 0.01, *p* < 0.01; R^2^ = 0.208, *p* < 0.01, *p* < 0.01; and day 7, R^2^ = 0.137, *p* < 0.01), and PC (day 1, R^2^ = 0.124, *p* < 0.01; day 3, R^2^ = 0.145, *p* < 0.01; day 5, R^2^ = 0.178, *p* < 0.01; and day 7, R^2^ = 0.136, *p* < 0.01), which were negatively correlated with age. In this study, the AT and α2PI were significantly lower in the nonsurvivors than in the survivors. Because the mean age of the nonsurvivors was significantly higher than that of the survivors, these results may have been influenced to some extent by age.

Patients with severe COVID-19 exhibited hypercoagulability. Coagulation biomarkers, such as PT-INR, fibrinogen, D-dimer, and FDP, were elevated in patients whose conditions were aggravated by COVID-19 after admission [22]. The procoagulant profiles in ICU patients with acute respiratory failure were characterized by a shorter clot formation time and increased clot strength, compared with healthy controls [23,24]. These studies also confirmed high fibrinogen and D-dimer levels. Fibrinolysis inhibition among COVID-19 patients, as indicated by the plasmin–antiplasmin complex (PAP)/plasminogen activator inhibitor 2 (PAI-2) ratio, was higher in survivors than in nonsurvivors, and a high D-dimer level and complete lack of clot lysis was reported in critically ill patients [25,26]. We also observed hypercoagulation and hyperfibrinolysis in COVID-19 patients. However, several markers that were mentioned in other reports as being biomarkers of COVID-19 severity (PLT, PT-INR, APTT, fibrinogen, AT activity, α2PI, PC, and PS) fell within the normal ranges in the present study. These findings also agree with those of other reports. For example, APTT, PT-INR, PLT, and AT activity reportedly showed no dramatic changes and fell within the normal ranges during the 7 days after admission [27]. Only tPAPAI-1C, as mentioned in the present report, was dramatically higher in COVID-19 nonsurvivors during the 7 days after admission.

Patients who suffer from DIC are generally assumed to exhibit hypercoagulation and hyperfibrinolysis. In fact, 8.4% of the enrolled patients and 18.2% of the nonsurvivors had DIC in this study. Martín-Rojas et al. also reported that the prevalence of overt DIC was 5.3% overall among COVID-19 patients, 22.2% among nonsurvivors, and 3.7% among survivors [28]. The most common parameters associated with sepsis-induced DIC are a prolonged PT-INR, a low PLT count, a low fibrinogen level, low AT activity, and a low PC level. The values of these biomarkers are often outside the reference ranges, suggesting a systemic coagulation abnormality. However, systemic coagulopathy reportedly occurs in severe sepsis patients without COVID-19, but not in COVID-19 patients [13,27,29,30]. Postmortem specimens of peripheral lung tissues from COVID-19 patients were described as exhibiting diffuse alveolar damage, severe endothelial injury, and widespread thrombosis with microangiopathy [31]. Hence, COVID-19 patients might experience severer regional pulmonary coagulopathy than systemic coagulopathy, compared with sepsis patients.

Our study suggested that vWF, TM, and tPAPAI-1C were higher among nonsurvivors than among survivors. The present study indicated that the maximum tPAPAI-1C value (OR = 1.034; 95% CI, 1.014–1.061; *p* = 0.0041) was an independent predictor of mortality in COVID-19 patients. t-PA and PAI-1 are mainly produced by endothelial cells, and tPA is regulated by PAI-1. tPA binds to PAI-1 on endothelial cells and in plasma to form tPAPAI-1C. An elevated serum tPAPAI-1C level suggests hyperfibrinolysis and endothelial damage. A relation between endothelial damage and the severity of COVID-19 has been previously reported. Vassiliou et al. demonstrated that the levels of soluble (s) E-selectin, sP-selectin, angiopoietin-2, and soluble intercellular adhesion molecule 1 (sICAM-1) were higher in nonsurvivors than survivors [32]. Another study revealed that vWF antigen and soluble TM were associated with mortality [33]. These factors are known biomarkers of endothelial damage. On the other hand, a previous study showed that increased tPAPAI-1C was strongly correlated with the PAI-1 activity level, indicating the inhibition of the fibrinolytic system [34]. Jin et al. reported that tPAPAI-IC and D-dimer were independent risk factors for mortality in COVID-19 patients [35]. Their patient population contained 105 patients with mild disease, 18 with severe disease, and 24 with critical disease. Of the 147 patients in total who were enrolled in the study, 10 patients were nonsurvivors. In our study, all 164 enrolled patients had severe or critical conditions, and 33 patients were nonsurvivors; thus, the overall severity was much higher in the presently reported study.

These findings suggest that COVID-19 patients with a poor outcome exhibited not only systemic coagulopathy, but also an impaired fibrinolytic system and endothelial damage. The measurement of tPAPAI-1C may be useful for providing appropriate treatment to COVID-19 patients suspected of having a poor prognosis.

Our study had several limitations. First, because this study was a retrospective observational study performed at a single center, the number of patients was relatively small, and the coagulation parameters were only measured every other day for 7 days after admission. Additionally, a higher dose of heparin was administered to nonsurvivors who had been undergoing ECMO and CRRT. Thus, the cause of the prolonged APTT may be the high-dose heparin infusion. A multicenter, prospective study conducted with a larger sample size and patients covering a wider range of disease severity, including asymptomatic or mild infection, and daily measurements of the hematological parameters is needed. 

## 5. Conclusions

This study demonstrated that the activation of coagulation and the impairment of the fibrinolytic system, along with endothelial damage, are deteriorating factors affecting the prognosis of severe or critical COVID-19 patients. In particular, elevated tPAPAI-1C could be a useful predictor of a poor patient outcome.

## Figures and Tables

**Table 1:** Patient characteristics.

Characteristics		Total (*n* = 164)	Survivors (*n* = 131)	Nonsurvivors (*n* = 33)	*p* Value
Age in years mean ± SD		62.5 ± 12.9	61.0 ± 13.2	69.1 ± 9.5	0.0023
Sex	Male, *n* (%)	117 (71.3)	98 (74.8)	19 (57.6)	0.0561
	Female, *n* (%)	47 (28.7)	33 (25.2)	14 (42.4)	
BMI, median (IQR)		24.8 (22.8–28.1)	24.9 (22.9–27.8)	23.9 (21.8–29.2)	0.7568
History of smoking, *n* (%)		86 (52.4)	68 (51.9)	17 (51.5)	1.0000
Charlson comorbidity index, median (IQR)		1 (0–2)	1 (0–2)	1.5 (1–3)	0.0016
APACHE II, median (IQR)		11 (7–17)	9 (6–14.3)	16 (13–20.5)	<0.0001
SOFA score; median (IQR)		3 (2–6)	2 (2–5)	7 (5.3–9)	<0.0001
ISTH overt DIC (≥5 points), *n* (%)		8 (4.9)	2 (1.5)	6 (18.2)	0.0009
Severity	Severe, *n* (%)	84 (51.2)	82 (62.6)	2 (6.1)	<0.0001
	Critical, *n* (%)	80 (48.8)	49 (37.4)	31 (93.9)	<0.0001

Values are presented as the mean ± standard deviation, median with interquartile range (IQR), or number (%). BMI: body mass index, APACHE II score: acute physiology and chronic health evaluation II score, SOFA: sequential organ failure assessment score, ISTH: International Society of Thrombosis and Hemostasis; DIC: disseminated intravascular coagulation.

**Table 2:** Comorbid conditions in patients.

Comorbidities	Total (*n* = 164)	Survivors (*n* = 131)	Nonsurvivors (*n* = 33)	*p* Value
Hypertension, *n* (%)	78 (47.6)	58 (44.3)	20 (60.6)	0.1189
Diabetes, *n* (%)	66 (40.2)	50 (38.2)	16 (48.5)	0.3229
Chronic heart disease, *n* (%)	32 (19.5)	23 (17.6)	9 (27.3)	0.2236
Chronic lung disease, *n* (%)	29 (17.7)	21 (16.0)	8 (24.2)	0.3080
Stroke, *n* (%)	16 (9.8)	10 (7.6)	6 (18.2)	0.0956
Chronic kidney disease, *n* (%)	13 (7.9)	8 (6.1)	5 (15.2)	0.1399
Malignancy, *n* (%)	11 (6.7)	6 (4.6)	4 (12.1)	0.1163
Chronic liver disease, *n* (%)	11 (6.7)	7 (5.3)	4 (12.1)	0.2341
Immunosuppressive disease, *n* (%)	10 (6.1)	8 (6.1)	2 (6.1)	1.0000

**Table 3:** Patient treatments.

Treatments	Total (*n* = 164)	Survivors (*n* = 131)	Nonsurvivors (*n* = 33)	*p* Value
Mechanical ventilation, *n* (%)	81 (49.4)	50 (38.2)	31 (93.9)	<0.0001
High-flow nasal canal, *n* (%)	63 (38.4)	53 (40.5)	10 (30.3)	0.3220
Extracorporeal membrane oxygenation, *n* (%)	10 (6.1)	5 (3.9)	5 (15.2)	0.0291
Renal replacement therapy, *n* (%)	16 (9.8)	3 (2.3)	13 (39.4)	<0.0001
Prone position, *n* (%)	66 (40.2)	38 (29.0)	28 (84.9)	<0.0001
Corticosteroids, *n* (%)	158 (96.3)	128 (97.7)	30 (90.9)	0.0965
Remdesivir, *n* (%)	84 (51.2)	73 (55.7)	11 (33.3)	0.0312
Tocilizumab, *n* (%)	82 (50.0)	63 (48.1)	19 (57.6)	0.4363
Heparin, *n* (%)	164 (100)	131 (100)	33 (100)	1.0000
Use of antibiotics in secondary infections, *n* (%)	80 (48.8)	49 (37.4)	31 (93.9)	<0.0001

**Table 4:** Comparison of maximum or minimum parameter values between survivors and nonsurvivors.

Parameters	Reference Range	Survivors (*n* = 131)	Nonsurvivors (*n* = 33)	*p* Value
WBC (/µL)	4000–9000	11,420 (9110–14130)	13,610 (9710–15138)	0.1092
CRP (mg/dL)	≤0.3	9.4 (5.5–14.5)	11.0 (6.4–21.8)	0.2045
PLT (×10^4^/µL)	15–35	19 (16–25)	14 (9–19)	0.0006
PT-INR	0.85–1.15	1.15(1.07–1.26)	1.25 (1.13–1.52)	0.0024
APTT (sec)	26.1–35.6	37.8 (32.5–47.2)	57.0 (42.9–97.1)	<0.0001
Fibrinogen (mg/dL)	150–450	342 (284–429)	270(221–353)	0.0022
SF (µg/mL)	<7	10.2 (6.1–22.6)	23.3 (10.2–62.1)	0.0004
TAT (ng/mL)	≤3	6.8 (4–13.8)	13.6 (8.0–20.2)	0.0023
vWF (%)	50–155	380(326–444)	399 (359–507)	0.0284
α2PI (%)	85–115	109 (99–117)	92 (85–109)	0.0003
PIC (µg/mL)	≤0.8	2.1 (1.6–2.8)	1.9 (1.3–2.9)	0.1495
D-dimer (µg/mL)	≤0.5	2.0 (1.1–3.7	5.9 (3.3–13.1)	<0.0001
FDP (µg/mL)	≤5	5.5 (5.0–10.5)	13.4 (9.0–29.8)	<0.0001
AT activity (%)	80–120	80 (69–89)	69 (57–81)	0.0001
TM (U/mL)	12.1–24.9	11.5 (4.2–18.6)	18.8 (5.5–27.4)	0.0061
PC (%)	70–150	84 (68–109)	70 (51–84)	0.0044
PS (%)	73–137	97 (85–109)	86 (70–96)	0.0038
tPAPAI-1C (ng/mL)	0–50	54.0 (39.0–75.0)	84.5 (68.0–130.5)	<0.0001

Values are presented as the mean ± standard deviation, median with interquartile range (IQR), or number (%). CRP: c-reactive protein, PLT: platelet counts, PT-INR: prothrombin time–international normalized ratio, APTT: activated partial thromboplastin time, SF: soluble fibrin, TAT: thrombin/antithrombin III complex, vWF: von Willebrand Factor, α2PI: α2plasmin inhibitor, PIC: plasmin/α2plasmin inhibitor complex, FDP: fibrin/fibrinogen degradation products, AT activity: antithrombin activity, TM: thrombomodulin, PC: protein C, PS: protein S, tPAPAI-1C: tissue plasminogen activator/plasminogen activator inhibitor-1 complex.

## Data Availability

The data presented in this study are available from the corresponding author on reasonable request. The data are not publicly available due to patients’ privacy.
